# Patient experience of centralized acute stroke care pathways

**DOI:** 10.1111/hex.12685

**Published:** 2018-03-31

**Authors:** Catherine Perry, Iliatha Papachristou, Angus I.G. Ramsay, Ruth J. Boaden, Christopher McKevitt, Simon J. Turner, Charles D.A. Wolfe, Naomi J. Fulop

**Affiliations:** ^1^ Alliance Manchester Business School University of Manchester Manchester UK; ^2^ Department of Applied Health Research University College London London UK; ^3^ Department of Primary Care and Public Health Sciences Kings College London London UK; ^4^ National Institute of Health Research Comprehensive Biomedical Research Centre Guy's and St Thomas’ NHS Foundation Trust King's College London London UK; ^5^ Centre for Primary Care Division of Population Health Health Services Research and Primary Care School of Health Sciences, Faculty of Biology, Medicine and Health, University of Manchester Manchester UK; ^6^ National Institute of Health Research, Collaboration for Leadership in Applied Health Research and Care South London London UK

**Keywords:** centralization of services, patient/carer experience, stroke care

## Abstract

**Background:**

In 2010, Greater Manchester (GM) and London centralized acute stroke care services into a reduced number of hyperacute stroke units, with local stroke units providing on‐going care nearer patients’ homes.

**Objective:**

To explore the impact of centralized acute stroke care pathways on the experiences of patients.

**Design:**

Qualitative interview study. Thematic analysis was undertaken, using deductive and inductive approaches. Final data analysis explored themes related to five chronological phases of the centralized stroke care pathway.

**Setting and participants:**

Recruitment from 3 hospitals in GM (15 stroke patients/8 family members) and 4 in London (21 stroke patients/9 family members).

**Results:**

Participants were impressed with emergency services and initial reception at hospital: disquiet about travelling further than a local hospital was allayed by clear explanations. Participants knew who was treating them and were involved in decisions. Difficulties for families visiting hospitals a distance from home were raised. Repatriation to local hospitals was not always timely, but no detrimental effects were reported. Discharge to the community was viewed less positively.

**Discussion and conclusions:**

Patients on the centralized acute stroke care pathways reported many positive aspects of care: the centralization of care pathways can offer patients a good experience. Disadvantages of travelling further were perceived to be outweighed by the opportunity to receive the best quality care. This study highlights the necessity for all staff on a centralized care pathway to provide clear and accessible information to patients, in order to maximize their experience of care.

## 1 INTRODUCTION

### 1.1 Centralized acute stroke care pathways

There is evidence to support the centralization of many specialist hospital services, with service provision concentrated in a reduced number of sites.^1^ During recent years in various countries, acute stroke care services have been centralized into specialist centres, in order to improve access to inpatient stroke care.^2^ In England, this is in response to the National Stroke Strategy^3^ which identified care in a stroke unit as the biggest single factor that could improve outcomes.

**Figure 1:**
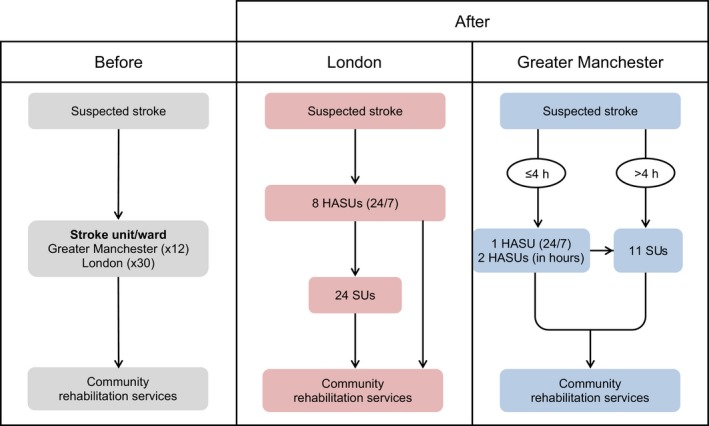
Centralized acute stroke care services in Greater Manchester and London. Source: Morris et al.^2^ Key: HASU—hyperacute stroke unit. SU—stroke unit

In 2010, Greater Manchester (GM) and London centralized acute stroke care services into a reduced number of hyperacute stroke units (HASUs), designed to provide all necessary evidence‐based care within 72 hours of onset of stroke. Patients were then repatriated as necessary to local stroke units, which provided on‐going care nearer patients’ homes. Referral pathways differed: in GM, patients reaching hospital within 4 hours of symptoms commencing were eligible for HASU care, with those presenting later admitted to local stroke units; in London, all suspected stroke patients were eligible (Figure 1). The intention of centralizing services was to reduce mortality and morbidity by addressing variations in provision of evidence‐based care.^4^ The centralization in GM and London was associated with different outcomes. London patients were significantly more likely to receive evidence‐based clinical interventions than GM patients, as a greater proportion of London patients were treated at a HASU.^5^ Length of hospital stay was reduced in both GM and London; however, only in London was stroke mortality significantly reduced compared to other urban areas of England.^2^ Fulop et al^6^ identify provision of evidence‐based care and clinical outcomes as two components of a conceptual framework for the analysis of major system change (such as centralization). This study addresses another key component in the framework, that of patient experience.

The impact of centralized acute stroke care pathways on patient experience has not been explored in depth. Centralized services may affect patient experience in several ways. Services are likely to be relatively high volume, and patient satisfaction with stroke services has been reported as lower in larger stroke services.^7^ Care may be provided in an unfamiliar environment with travelling distances increased for patients and families.^8^ Payne et al^9^ reported that travel for cancer treatment had been described as inconvenient and could be perceived as a barrier to treatment. However, when studying angioplasty services, Sampson et al^10^ concluded that although inconvenient, people would travel further to access centralized services. A survey of the experience of patients and carers of the newly centralized stroke care pathways in London reported that the majority of stroke patients and carers were either “happy” or “did not mind” being treated in a more distant HASU, and although concern was expressed about repatriation, only 6% reported any negative effect of the transfer.^8^

### 1.2 The importance of patient experience

The definition of quality in health care has expanded to include patient experience,^11^ and the concept is prominent in the measurement of health service performance.^12^ Although there is no universal definition of patient experience,^11^ many definitions reflect that of the Kings Fund Point of Care Programme: “the totality of events and interactions that occur in the course of episodes of care.”^13^ Patient experience is more than “patient satisfaction,” and asking patients “what happened” during an episode of care is more valid in judging quality of care than just asking about “satisfaction.”^14^ It is mandatory for NHS providers to gather patient experience data,^15^ and understanding how patients experience care can highlight substandard care.^16^ In 2012, the National Institute for Health and Care Excellence^17^ produced a quality standard to provide the NHS with clear commissioning guidance on the components of a good patient experience: 14 quality statements against which patients’ experience can be measured (Table 1).

**Table 1:** National Institute for Health and Care Excellence quality standard for patient experience (2012)^17^

Statement 1	Patients are treated with dignity, kindness, compassion, courtesy, respect, understanding and honesty.
Statement 2	Patients experience effective interactions with staff who have demonstrated competency in relevant communication skills.
Statement 3	Patients are introduced to all health‐care professionals involved in their care and are made aware of the roles and responsibilities of the members of the health‐care team.
Statement 4	Patients have opportunities to discuss their health beliefs, concerns and preferences, to inform their individualized care.
Statement 5	Patients are supported by health‐care professionals to understand relevant treatment options, including benefits, risks and potential consequences.
Statement 6	Patients are actively involved in shared decision making and supported by health‐care professionals to make fully informed choices about investigations, treatment and care that reflect what is important to them.
Statement 7	Patients are made aware that they have the right to choose, accept or decline treatment, and these decisions are respected and supported.
Statement 8	Patients are made aware that they can ask for a second opinion.
Statement 9	Patients experience care that is tailored to their needs and personal preferences, taking into account their circumstances, their ability to access services and their coexisting conditions.
Statement 10	Patients have their physical and psychological needs regularly assessed and addressed, including nutrition, hydration, pain relief, personal hygiene and anxiety.
Statement 11	Patients’ experience continuity of care delivered, where possible, by the same health‐care professional team throughout a single episode of care.
Statement 12	Patients experience coordinated care with clear and accurate information exchange between relevant health‐care and social care professionals.
Statement 13	Patients’ preferences for sharing information with their partner, family members and/or carers are established, respected and reviewed throughout their care.
Statement 14	Patients are made aware of who to contact, how to contact them and when to make contact about their on‐going health‐care needs.

## 2 BACKGROUND TO THIS STUDY

### 2.1 What is already known about patient experience of acute stroke care pathways?

In the absence of specific data on patient experience of acute stroke care pathways in GM and London prior to the centralization, the existing literature was used to help frame this analysis. The literature provides evidence in relation to the stages of the acute stroke care pathway and also to cross‐cutting issues that relate to all stages of care.

### 2.2 Initial transfer to hospital

For the majority of stroke patients (70%), first point of contact with services is through the emergency medical services,^18^ and research suggests that patients and carers have a generally positive experience with these teams.^19^, ^20^ Those calling the emergency services found call handlers to be reassuring and calming,^18^, ^19^ although not all were clear whether an ambulance was on the way or when it might arrive.^18^ Along with speed of arrival of assistance,^19^ the importance to patients of “holistic care” from the emergency medical services (defined as handling the whole situation, not just the person with the symptoms) was highlighted.^20^

### 2.3 In‐hospital care

Studies of inpatient hospital stroke care indicate that, overall, people had a positive experience.^21^, ^22^, ^23^, ^24^, ^25^, ^26^ Patients treated on acute stroke units were generally more satisfied with their care than those on general wards,^21^, ^27^, ^28^ Often however, appreciation of a service as a whole was tempered by concerns about service shortfalls,^29^ particularly in relation to initial experience of inpatient care, the provision of therapy and general aspects of care.

In an interview study of people admitted to stroke units, although many reported fast access to assessment on admission to the Accident and Emergency Department (A+E), others described delays because of poor availability of staff or beds, and perceived that stroke was not treated as a medical emergency.^19^ As patients and carers were generally aware of the importance of time to treatment, these delays caused anxiety and frustration. Those admitted “out of hours” reported poor availability of some specialist services such as medical input and imaging, which some perceived as hindering their access to appropriate treatment.^19^

Lack of therapy (physiotherapy/speech therapy/occupational therapy) during inpatient care was reported.^29^, ^30^, ^31^ Some stroke patients associated this with their experience of setbacks in recovery.^29^ A lack of help in hospital with emotional problems, such as confusion or depression, has also been reported,^21^ resulting in a poorer experience of care, with depression a possible independent predictor of poor long‐term functional outcome after stroke.^32^

Most stroke patients have reported that they were always treated with respect and dignity,^21^, ^23^ although other studies have indicated that stroke patients did not always receive the help that they needed with general activities such as eating or washing.^21^, ^31^ Carers felt that they needed to compensate for perceived shortfalls in the care of their relatives on occasion, although the general institutional nature of much hospital care was experienced as preventing family from participating in aspects of care.^24^, ^29^

### 2.4 Discharge home

Discharge preparation has been described as lacking in the past and more recently.^21^, ^23^, ^25^, ^26^, ^30^ Ellis‐Hill et al^33^ explored what constituted a “good” or “poor” experience in the transition from hospital to home through interviews with 20 stroke patients and 13 carers. Discharge was perceived to be successful by stroke patients if they maintained a sense of momentum about their recovery, felt supported and felt informed about what was happening to them. In the Healthcare Commission survey,^21^ although 90% of people thought that their GP had been given sufficient information to care for them once at home and most patients (63%) reported that all the services they needed after leaving hospital were arranged, 15% said that such services were not arranged. Those who had been cared for on a specialist stroke ward were more likely to report that services had been arranged than those who had not.^21^

### 2.5 Information provision

Receiving adequate information about care contributes to a positive experience for stroke patients and carers,^28^ for example by reducing anxiety.^29^ Varied experiences of information provision whilst in hospital have been described. Some considered they had received enough information, others felt that they were overloaded, or that they had not received enough,^23^, ^24^, ^25^, ^29^, ^34^, ^35^ indicating the need for a service responding to differing patient needs. Payne et al^34^ identified that families of stroke patients found it difficult to get time with staff to find out about a patient's care. Where a lack of information was perceived, this was particularly in relation to treatment, and what care to expect after discharge.^29^

### 2.6 Personalized care

When asked what constituted good stroke care, patients articulated that being personally valued and cared about by health‐care staff was important.^24^ This was echoed by Hewitt et al^28^ in their interviews with 50 patients and 33 carers in acute, inpatient rehabilitation and community phases of care, who reported that being treated with individual care and attention, and having trust and confidence in health‐care professionals, led to a positive experience of care. Morris et al^29^ also reported that stroke patients wanted health‐care staff to see them in context as people, not just patients, as this improved their experience.

### 2.7 Study aim and objectives

The aim of the study reported here was to analyse in depth the impact of the GM and London centralized acute stroke care pathways on the experience of patients. Reflecting both the literature on patient experiences of acute stroke care and our knowledge of the centralized acute stroke care pathways, the specific objectives were to explore experiences of:

1. initial contact with the emergency care services and transfer to hospital;
2. reception at hospital, whether stroke was treated as a medical emergency;
3. in‐hospital care, particularly in relation to admission to a more distant HASU;
4. repatriation to local stroke unit;
5. discharge home, particularly if from a more distant HASU;
6. provision of information across the care pathway.

## 3 STUDY METHODS

### 3.1 Sample

Patients were recruited from 3 case study sites in GM (the sole 24/7 HASU, one of two in‐hours HASUs, one of ten local stroke units) and 4 sites in London (two of eight 24/7 HASUs, two of 24 local stroke units). Any patient diagnosed with stroke was eligible for inclusion provided they had adequate cognitive function, determined by their ability to give informed consent to participate. Sampling was purposive, that is deliberately non‐random, to select those in the best position to act as key informants.^36^ A maximum variation strategy was employed^37^ in order that a range of experience of the centralized pathway was represented: admission to a HASU; admission to a local stroke unit (GM); discharge from a HASU; repatriation from HASU to local stroke unit; discharge from a local stroke unit. The sample was also selected to include males and females and a range of ages.

### 3.2 Participant recruitment and data generation

Recruitment and data generation occurred between April 2013 and May 2016. Potential participants approached shortly before discharge from hospital by a research nurse or clinician were given a study information sheet, and asked whether they would speak to a researcher. The researcher explained the study, and if willing to participate permission to contact patients after their discharge was obtained.

Patients were interviewed at home within 3 months of discharge, with fully informed written consent. Carers were included if the patient wished, or they were incidentally available at the time and the patient was agreeable for them to contribute: they were asked about their perceptions of care received by the stroke patient. Semi‐structured interviews were used as they offer a good way to generate data regarding individuals’ experiences and emotions.^38^ A semi‐structured interview schedule was developed (Data S1), with reference to the literature reviewed, established recommendations such as the NICE quality standards for patient experience^17^ and in relation to the new care pathways. A patient co‐investigator assisted with development of the schedule, which was also discussed with the Study Steering Committee (including patient representatives) and a stroke patients' research group. Interview questions defined the area to be explored,^39^ but allowed interviewer or interviewee to diverge in order to follow up particular areas in more detail.^40^ With the permission of participants, interviews were digitally audio‐recorded, and then professionally transcribed.

### 3.3 Data analysis

Interview transcripts were uploaded onto NVivo software to aid data management.^41^ A thematic analysis^42^ was undertaken, initially using a deductive approach guided by a baseline framework developed from the literature (Table 2), as in template analysis.^43^ Using the framework sensitized researchers to elements in the data that might otherwise have been missed.^44^ As analysis continued, an inductive approach was used, transcripts were coded, and themes were collated as described by Bradley et al.^42^ The final data analysis framework was developed with themes organized under the five chronological phases of the centralized stroke care pathway (Table 3).

**Table 2:** Baseline framework used for data analysis (from literature)

Main themes	Subthemes
Responding to stroke symptoms	Onset of stroke symptoms Barriers to contacting emergency services Benefits of contacting emergency services
Ambulance service	Timely transportation Impact of paramedic communication Pre‐hospital information and diagnosis
Explanation and information	Transparency of health‐care professionals Meeting expectations with hospital treatments Carer's role in decision making
Person‐centred approach	Taking a personal interest in the patient's well‐being Feelings of isolation
Availability of therapy	Insufficient physiotherapy/speech therapy Meeting on‐going aftercare needs

**Table 3:** Final data analysis framework

Phases of stroke care pathway	Themes
Initial transfer to hospital	Timely response Information about likely diagnosis Concerns about transfer to HASU
Reception at hospital	Timely investigations and treatment Stroke—a medical emergency
In‐hospital care	Clear explanations and shared decision making Known staff Difficulties for families—travel to more distant HASU Consideration from staff concerning travel
Repatriation to local hospital	Staff uncertainties Delay in obtaining bed at local stroke unit Transportation to local unit Transfer of care to local unit
Discharge home	Communication with GPs Continuation of therapy and follow‐up

HASU, hyperacute stroke units.

Steps were taken to enhance methodological rigour. To ensure dependability,^45^ two people (CP/IP) used the baseline framework to analyse early interviews, with some transcripts analysed by both to ensure consistency in data coding. The emerging inductive analysis was discussed with a subgroup of the authors (AIGR, NJF, CM, RJB). To enhance credibility,^45^ interim versions of the analysis were presented to stroke patient support groups who were asked whether the findings (and our interpretation of them) reflected their own experiences and/or made sense to them. These patients agreed that the findings made sense.

### 3.4 Ethical approval

Ethical approval was received in September 2011 from the London East NHS Research Ethics Committee (Ref 11/LO/1396).

## 4 FINDINGS

There were 36 stroke patients in the sample (17 F, 19 M, aged 38‐90 years), along with 17 partners or carers. A range of experiences were represented in terms of whether people were admitted to a HASU or a local stroke unit, were transferred internally or were repatriated to a local stroke unit (Table 4). Findings are presented in relation to the five chronological phases of the centralized stroke care pathway (Table 3).

**Table 4:** Participant details

Hospital	Sex		Age range	No. of carers participating	Care pathway followed	No.
	M	F				
London A	3	2	38‐86	3	All care at HASU HASU‐local unit	2 3
London B	4	1	58‐83	1	HASU‐local unit Out of area‐local unit All care at local unit	2 2 1
London C	3	3	51‐86	2	All care at HASU HASU‐HASU stroke unit	4 2
London D	1	4	72‐90	3	HASU‐local unit	5
GM F	1	3	41‐82	0	All care at HASU HASU‐HASU stroke unit	2 2
GM G	2	0	55‐68	2	All care at HASU	2
GM H	5	4	52‐86	6	HASU‐local unit All care at local unit Local unit‐HASU‐local unit	5 3 1
Total	19	17	38‐90	17		

HASU, hyperacute stroke units; GM, Greater Manchester.

### 4.1 Initial transfer to hospital

Most people who experienced stroke were transported to hospital by ambulance. Participants reported that ambulances arrived quickly and ambulance staff gave clear information about likely diagnosis, which served to reduce anxiety: “They seemed professional, they seemed friendly you know, supportive you know, so I felt safe.” (London, patient). However, being told of by‐passing a local hospital to attend a more distant HASU caused concern: “We're going further, that's going to take longer, what happens if it gets worse on the way?” (GM, family member). The necessity for clear information to allay such fears was evident. One patient was reassured when told they would: “Go to the right place that would sort me out” (London, patient) and another stated: “They said we're taking you to (HASU) because they've got a specialist stroke unit there, effectively, and I said ‘well that's fine’.” (GM, patient). This can be contrasted with the experience of a woman who was transferred by ambulance from a local unit to a HASU, whose anxiety was increased by the apparent confusion of ambulance staff not able to explain what was happening: “I'd never been in an ambulance before, which was daunting in itself, and then the ambulance man was saying ‘Well we've not had a proper handover, we don't know what's going on’.” (GM, patient).

### 4.2 Reception at hospital

In the centralized care pathway stroke teams met the patient on arrival at A+E. Participants were impressed with this reception, perceiving that stroke was treated as a priority and a medical emergency: “You went in and they were so ready for him, I know they'd radio‐ed through, I know they were prepared for him.” (GM, family member); “You went through the doors and there's all these people standing there ready to … just waiting.” (London, patient). Participants reported receiving timely investigations, such as scans, and that the teams treating them knew what they were doing: “It felt [from] the initial entry some kind of action plan was very quick, and wasn't that we were going to be sit up in the corner somewhere, forgotten about for hours.” (GM, family member). The experience of an organized and timely reception was important in combatting anxiety: “Very reassuring….. because I was obviously panic stricken.” (London, patient).

### 4.3 In‐hospital care

Generally, participants indicated that they knew who was treating them, they received clear explanations and were involved in decisions about their care, which are all recognized quality standards for patient care.^17^ “If you asked a direct question you got a direct answer, and I think that was really important that you felt that you weren't being fobbed off.” (London, family member). However, with admission to a more distant HASU, visiting for families was raised as an issue: “It was a bit awkward being so far away.” (GM, patient); “I can imagine it would affect people if they were in Kent or something.” (London, patient). Carers recounted difficulties in visiting. One said: “It was so expensive….. I were there twice a day,” and also explained the impact that the distance to travel had: “Back home again, you have no time. I think I'd get home, took the dog out, come back and go again….. just no time and you couldn't just not go.” (GM, family member). Participants recounted efforts made to ameliorate these issues, one mentioning hospital staff being flexible about visiting times and another a grant that could be applied for to help with travel costs, although she had not done so: “You could put in for this grant. But I haven't been able to … I phoned the number and she sent me another leaflet and said… put in for it from your physiotherapist or your GP….. haven't took it any further.” (GM, family member).

### 4.4 Repatriation to local hospital

As part of the centralized stroke pathway, patients admitted to a HASU which was not their local hospital were returned to their local stroke unit after receiving their acute care, if they were not well enough for discharge home. For most participants in the study, this repatriation happened smoothly: “Once they told me yes there's a bed available they then came and said we've ordered an ambulance and it will take between one and 4 hours, I remember them saying that. But it came well within 4 hours, under 4 hours.” (GM, patient).

However, some difficulties were described. It was reported that hospital staff were not always sure which hospital a patient should be repatriated to, which may reflect that staff were learning to work with a new care pathway and had initial uncertainties: “Made a right pig's ear of it didn't she? Because she came back and apologized the following day…. She came back and said, ‘Oh I'm very sorry, I'm a stranger here, I know you live in [name]’, I said, ‘Yes’, ‘But yes you can go to [name of local unit], it's my mistake’.” (GM, patient). Other people described delays: “We were waiting for a bed to become available at (local unit), that was the reason he was in (HASU) a bit longer.” (GM, family member). Although this could be frustrating, patients generally accepted the situation if they were kept informed about what was happening. In contrast, another family member, who received unclear and conflicting information, felt annoyed and confused: “Somebody told us she would definitely be going at one time, then she didn't go and then somebody else said no, ……you know it was a little bit confusing.” (London, family member). Thus, the centrality of accurate information was emphasized again at this stage of the pathway: “They kept me informed of what was going on, that was….I think is the most important thing.” (London, patient).

Once a date and time for transfer were given, some delays in transport to a local stroke unit were described. This delay was not tolerated well by patients and their families: “We weren't very happy if you recall at the time with transfer from HASU to local stroke unit, because it took 6 hours, which left both of us in a very het up and upset state.” (London, family member). For some, delay in transportation to a local unit resulted in transfer happening later in the evening, which was another situation patients found unacceptable. One patient described his experience: “The next day they said they wanted to send me to [hospital] which was the nearest hospital to home. I set out, I didn't set out, they said you'll be going later on in the day…. Well I sat around all day and nothing happened and by half past nine at night no ambulance had arrived so I said ‘Well I'm not going, I'm not going to be carted in the middle of the night through a big city’.” (GM, patient). This patient described being transferred to a bed on another ward for one night because of the pressure on HASU beds, which for him was unsettling.

Most people perceived that their care was continued smoothly once they were transferred to their local stroke unit, that staff were aware of what had happened to them and that repatriation did not have any impact on the trajectory of their recovery: “It did feel like it was just a continuation of the treatment. It didn't feel like we'd been passed from one place to another…. They knew what had happened, they'd asked a few questions, but it wasn't like we had to start from scratch.” (GM, patient). Some commented favourably on the increase in therapy input once they had been transferred (an increase which would be expected as local units were focussed on rehabilitation).

### 4.5 Discharge home

With centralized acute stroke care pathways, some patients discharged home from a HASU would be discharged to a different area than that in which they had received their acute care. This potentially posed challenges to hospital teams who did not know the local processes of care, or the teams to whom they were discharging people. In terms of transfer between hospital and community, most participants thought that communication between hospitals and GPs happened effectively and that their GP was aware of their stroke: “Yes the GP got a letter from [HASU] before we got home, so although I took along my discharge note with me it wasn't actually necessary.” (GM, patient). However, some people were not clear about their follow‐up once home and were unsure about when, whether or how this was to happen, or experienced some delay. For example: “It's unclear even to me today what's going to happen with physiotherapy in the future because apparently there is…. a waiting list and I've not heard much from them.” (GM, patient).

## 5 DISCUSSION

This study explored patient experience of centralized acute stroke care pathways in two metropolitan areas. Similar experiences were reported by those from the two regions, which is perhaps unsurprising: although the care pathways differed in terms of who was eligible for HASU care (those presenting within 4 hours of symptom onset in GM/all patients in London), patients went through similar stages of care in both locations. The findings contribute to knowledge about patient experiences of centralized acute stroke care services and also to the wider body of knowledge relating to the centralization of services in general. The data demonstrate that patient experience can provide valuable information about how a service is operating, what is working well and what is not.^16^ For example, the patient observation that ambulance staff were not sure why they were transferring her to a different hospital indicates that appropriate information about care was not received. In addition, the value of talking to carers and family members is emphasized. Although not the focus of this study, they were able to elaborate on the impact of care received on the patient and themselves.

In terms of stroke‐specific findings related to the stages of the centralized care pathway, patients in this study were impressed with their contact with the emergency services, feeling reassured by their handling of the situation. The provision of reassurance has been identified as a key outcome for emergency ambulance services.^46^ Patients were also impressed with initial reception at hospital. Their experience of timely investigations and initial treatment suggests that stroke was treated as a priority and a medical emergency. This is in line with the National Stroke Strategy^3^ and in contrast to some earlier studies of non‐centralized pathways.^19^ Once admitted to hospital, patients described that they knew who was treating them, received clear explanations about their care and were involved in decisions, which are all recognized quality standards for patient care.^17^ This is again in contrast to much published literature^24^, ^29^, ^34^ and reflects what is known about the relationship between well‐organized stroke care and more positive patient experience.^14^ The extent to which timely investigation and treatments can be attributed to the centralized acute stroke care pathways is difficult to discern, as national initiatives such as the National Stroke Strategy^3^ were current at the time of the centralization in GM and London and would have driven such improvements in care. However, the centralization introduced HASUs, which are associated with a greater likelihood of receiving timely, evidence‐based care interventions.^5^

Other findings are relevant to the centralization of any service which involves patients being taken to more distant care settings and repatriated back to a local hospital. There is evidence in our data of these processes of care impacting upon patient experience. Patients, and particularly family members, expressed some disquiet on being informed that they were going further than their local hospital; and repatriation did not always happen in a timely manner (within 72 hours), resulting in patients feeling confused or anxious. It is at these points in the centralized care pathway that the importance of effective and timely information provision is emphasized. Clear explanations about the care pathway by the paramedic team, and being kept informed about when and where repatriation would happen by HASU staff, led to patients reporting a more satisfactory experience. This reflects the NICE quality standards for patient experience^17^ and the stroke‐specific literature in which the importance of clear information, communication and explanation about care is highlighted. The implications are that staff along all stages of a centralized care pathway need to be engaged with, and understand, the pathway of care and that information needs to be given to patients from the beginning of their care journey, before concerns and anxieties are expressed.

Difficulties for families visiting hospitals a distance from their homes were discussed, in terms of time and financial costs, but patients broadly prioritized quality of care and outcomes over the issues presented by being cared for at a more distant site. This is similar to the survey findings of Moynihan et al^8^ However, consideration could be given to how best to support patients and their families in this situation. Staff flexibility over visiting times and help with travel costs were both mentioned in this study. Officially extended visiting hours for those on centralized care pathways, or ensuring that visiting times coincide with the timing of public transport, as well as providing information about financial help available towards travel costs and assistance with making these claims, could improve patient and family experience.

Repatriation involved the transition of care from a HASU to a local stroke unit. The NICE quality standards^17^ suggest that care should be well coordinated between different health‐care professionals. The experience of patients in this study was that care was handed over smoothly, and nobody perceived that the transfer had any adverse effect on the trajectory of their recovery, similar to the findings of Moynihan et al^8^ However, one patient described being moved from a HASU to another ward for one night, before repatriation to a local stroke unit, because of pressure on HASU beds. This highlights how capacity issues need to be carefully considered in centralized services.

The most difficult transition for patients was discharge to the community, for example as evidenced by patients’ reports of not being clear about follow‐up care. Clarity about addressing on‐going care needs is one of the NICE patient quality standards.^17^ Although in general people being discharged from a specialist stroke ward are more likely to have adequate follow‐on care arranged than those from a general ward,^21^ patients in this study, who were all discharged from a specialist ward either at a HASU or local stroke unit, experienced some difficulties. This may reflect the focus of the stroke care pathway centralization on hyperacute care, and known variations in early supported discharge and community therapy services across GM and London. However, the centralized pathway resulted in patients being discharged into geographical areas remote from the specialist centre, where staff may have been unfamiliar with local discharge procedures. This issue would need to be addressed within any centralized service in order to ensure that care was carried on seamlessly in the community.

## 6 LIMITATIONS

There are a number of limitations to this study. First, only stroke patients who were cognitively able to participate in an interview were recruited into the study, and it is possible that the experience of those who had a less positive outcome after their stroke was different. This could have been addressed by actively recruiting family members of these patients to the study. Second, the study was of centralization of stroke care pathways in two metropolitan areas of England, centralized services in more rural areas may well be experienced differently by patients and carers. Third, some patients taken onto the centralized acute stroke care pathways in GM and London were ultimately not diagnosed with stroke. These so‐called “stroke mimics” were thus transferred to a hospital more distant from their homes with no particular benefit for themselves and were not part of this study. It is important that the experience of this group of patients is analysed in any overall evaluation of centralized acute stroke care pathways.

## 7 CONCLUSION

Patients on the centralized acute stroke care pathways in GM and London reported many positive aspects of care, and it is evident that they often experienced standards of care in line with the NICE quality standards.^17^ The findings suggest that the centralization of care pathways in general can offer patients a good care experience. The disadvantages of travelling further were perceived to be outweighed by the opportunity to receive the best quality care. The major contribution of this study is highlighting the necessity for all staff on a centralized care pathway to understand the patient journey and provide clear and accessible information to patients at every stage, in order to maximize their experience of care.

## CONFLICT OF INTERESTS

None.
