# Supplementary material for: Patient experience of centralized acute stroke care pathways
Source: Health Expect. 2018 Mar 31;21(5):909–18. doi: 10.1111/hex.12685 (PMC6186538; doi:10.1111/hex.12685)
Supplement: Supplementary file 1 [file HEX-21-909-s001.docx]

| Questions | Prompts | Info provided |
| --- | --- | --- |
| What happened when you had your stroke? | How and by whom were services contacted when you had your stroke? (999/NHS Direct/ GP/A&E/ambulance)  How long did it take before health services responded to your request for assistance?  How and by whom were you and your carer(s) told that you’d had a stroke?  Were you and your carer(s) supported by healthcare professional following the diagnosis of a stroke  Did you or your carer(s) feel confident that you were being treated by healthcare professionals who knew what to do?  Do you and or your carer(s) think you were admitted to hospital quickly enough?  Do you or your carer(s) feel your stroke was diagnosed quickly enough? Why/why not? | Communication, information – NICE quality statements Potential differences – e.g. London patients ALWAYS moved to a HASU in event of stroke; GM 4 hour window |
| What treatments did you receive? | e.g. thrombolysis/clot-busting drugs – scans?  Did healthcare professionals make sure you and your carer(s) understood throughout your treatment?  Were your healthcare options explained clearly to you and your carer(s), e.g. process, risks, benefits, at the right time?  Did you and carer(s) feel fully involved in the decisions being made? Were your views respected?  Did you and your carer(s) feel confident that the professionals overseeing your treatments were sufficiently knowledgeable and experienced in stroke healthcare? Did you and your carer(s) feel confident that the right decisions had been made? | Information provision, shared decision making, treatment with respect – NICE quality statements |
| How did people check how you were doing? What tests did you have? | e.g. physical, emotional, psychological – nutrition, hydration, swallowing, communication  Who carried out these checks? Were you confident that he/she knew what he/she was doing?  How often did they happen? Were they conducted by the same person each time? | Regular assessments, suitable expertise, continuity – NICE quality statements |
| What sorts of problems did you have?  Did you get enough help with any problems you might have had? | e.g. eating, swallowing, mobility, speech  Were there any specific healthcare needs that you or your carer(s) was not addressed by healthcare professionals?  Did healthcare professionals (e.g. doctors, nurses, therapists) introduce themselves to you and you carer(s) and explain what would happen to you?  Were you and your carer(s) satisfied and confident in the care you received? Did you feel staff were knowledgeable and competent in managing and delivering your post healthcare physical needs?  Do you and your carer(s) feel you were treated with dignity and respect? | Needs being addressed, dignity - NICE |
| Did you change ward when you were in hospital? | Were you admitted to stroke specific ward/unit? If not, were you and your carer(s) satisfied with the stroke specialist care you received?  How and by whom were you or your carer(s) informed about what was going to happen to you?  How and by whom was this explained to you and your carer(s)?  Did you or your carer(s) feel you had a say in your post stroke treatment? | Issues of transfer - e.g. HASU to SU in London; CSC/PSC to DSC in Greater Manchester |
| What happened when you were preparing to leave hospital? | How far in advance were you or your carer(s) informed that you would be leaving hospital?  Was there adequate time for you and your carer(s) to make arrangements for your on-going care?  Did you or carer(s) feel ready to leave/go home?  What concerns did you or your carer(s) have about your continued healthcare support at home?  Did you feel you or your carer(s) had a say in how your leaving hospital was to be arranged? (e.g. consenting to early supported discharge)  Did you or carer(s) feel suitably informed about sources of support once you had left hospital? (e.g. details in joint care plan - re community services, social services, stroke association)  What stroke related information were you and your carer(s) given on discharge and did this information include benefits you were entitled to (DLA, Blue Badge, Taxi Card, Freedom Pass etc)  Were you or your carer(s) given advice on how you might prevent future strokes? E.g. diet, smoking, exercise  Were you or your carer(s) given sufficient information about your medicines? (how to take, side effects) | Information provision, connection with other parts of patient pathway |
| What help have you needed since leaving hospital? Have you received this help? | e.g. for your emotional/psychological state of mind, physical abilities  Were services arranged for when you left hospital, e.g. physiotherapy, occupational therapy, speech therapy, mobility, social care?  Was information about your stroke, including medication needed for recovery shared with your GP?  Do you or your carer(s) know who to contact, in the event that you needed support?  How have community services/social services supported you?  Do you feel your needs have been met? | Ongoing support post hospital |
| Reflections | Was there anything particularly good about your stroke care in hospital and in the community?  Was there anything that could be improved, or that you felt was missing in your stroke care in hospital and in the community? |  |
| Confirm demographic info | Year of birth, hospital(s) |  |
